# Carpal Tunnel Release: A Four-Specialty Comparison Demonstrating Equal Clinical and Economic Efficacy

**DOI:** 10.1016/j.jhsg.2025.100863

**Published:** 2025-10-31

**Authors:** Andy M. Liu, Vikranth Mirle, Cody Lee, Jennifer Moriatis Wolf, Jason Strelzow

**Affiliations:** ∗ Department of Orthopaedic Surgery and Rehabilitation Medicine, University of Chicago, Chicago, IL; † Department of Orthopaedic Surgery, Columbia University, New York, NY; ‡ Department of Orthopaedic Surgery, Washington University in St. Louis, St. Louis, MO

**Keywords:** Carpal tunnel, Costs, General surgery, Hand surgery, Neurosurgery, Orthopaedics, Plastics

## Abstract

**Purpose:**

Carpal tunnel release (CTR), the most common hand surgery procedure in the United States, is routinely performed by orthopedic surgeons, neurosurgeons, plastic surgeons, and general surgeons. There is limited literature comparing clinical costs and research utilization among specialties. This study sought to determine treatment utilization, variations in cases, and cost trends among orthopedic, plastic, general, and neurosurgeons.

**Methods:**

A national insurance database was queried for patients who underwent open or endoscopic CTR between the years 2007 and 2022. Four cohorts based on provider specialty, orthopedic, plastic, general, and neurosurgery, were matched using the following factors: age, sex, diabetes, obesity, tobacco use, location of procedure, and Elixhauser Comorbidity Index. Average cost by specialty was then calculated and compared. Rates of therapy within 3 months after surgery and EMG and nerve conduction velocity studies within 3 months before the procedure were also compared.

**Results:**

The matched cohorts consisted of 13,107 patients each. Plastic surgeons and neurosurgeons had the highest average cost ($2,923.29 and $2,922.58), followed by orthopedic surgeons ($2,765.95), with general surgeons having the lowest cost ($2,607.02). Rates of preoperative EMG and nerve conduction studies were highest with neurosurgeons (28.5%), followed by orthopedics and plastics (22.6% and 23.4%, respectively), with general surgeons ordering the fewest (19.7%). For complications, general surgeons and neurosurgeons had a small but statistically higher infection rate (0.8%, 0.7% respectively) compared with orthopedic and plastic surgeons (0.5%, 0.5%).

**Conclusions:**

The current study demonstrates that overall orthopedic, plastic, neurosurgery, and general surgeons perform CTRs with similar overall costs and with similar postoperative complications.

**Type of study/level of evidence:**

Economic/decision analysis III.

Carpal tunnel syndrome (CTS) is one of the most common nerve entrapment syndromes, causing pain and numbness in the affected arm. It is estimated that between 4% and 5% of the population is affected with CTS, with a predominance in people aged 40–60 years.^1^^,^^2^ Although there is a range of conservative treatments available, the gold standard for surgical management is carpal tunnel release (CTR).^3^ Carpal tunnel release is a routine procedure in the United States, with evidence demonstrating improvement in quality of life after surgery.^4^^,^^5^

Between 400,000 and 500,000 surgeries are performed annually, with costs estimated at more than $2 billion.^6^ Carpal tunnel release surgery is regularly performed by surgeons from orthopedic, plastic, general, and neurosurgery backgrounds. Given the frequency of CTR, there is a litany of studies comparing the cost and outcomes of these procedures based on technique, procedure location, and patient demographics.^7^, ^8^, ^9^, ^10^ The classic technique used is open CTR, with endoscopic CTR and other minimally invasive techniques more recently described.^11^^,^^12^ Carpal tunnel release can be performed in the main operating room, ambulatory surgery center, or procedure room, with the operating room being less efficient and between two to four times as expensive as other sites, based on the technique.^13^

Despite the large body of studies on the cost of CTR based on technique and procedure location, there are minimal data regarding the cost of CTR based on surgeon specialty.^6^, ^7^, ^8^, ^9^, ^10^, ^11^, ^12^, ^13^ In this study, we aimed to determine the costs associated with primary CTR procedures among orthopedic, plastic, general, and neurosurgery specialties.

## Methods

### Database

A national insurance database, PearlDiver, consisting of 152 million insurance claims, from patients covered by Medicare, Medicaid Advantage, private insurance, and/or cash claims, was analyzed using International Classification of Disease codes (International Classification of Disease-9 and International Classification of Disease-10) and current procedural terminology (CPT) procedure codes. The database runs through Humana Health Insurance (PearlDiver Inc).

### Patient inclusion and exclusion criteria

Current procedural terminology codes for CTR (CPT-64721 and CPT-29848) were used to identify patients who underwent CTR from the years 2007–2022. Patients were filtered to only include initial CTR performed, excluding revision cases. Patients were required to remain active in the database for both 3 months prior to and after the procedure to ensure that patients who lacked adequate follow-up or switched insurance carriers were excluded. Patients with a prior upper-extremity burn within 2 months, fasciotomy within 2 months, or a distal radius fracture diagnosis on or within 2 weeks prior to their CTR procedure were also excluded to minimize confounding variables. Patients were separated into cohorts based on provider specialty (orthopedic surgeons, plastic surgeons, general surgeons, or neurosurgeons). Patients without a provider specialty identified were also excluded. The four cohorts were then matched for common comorbidities, including age, sex, Elixhauser Comorbidity Index, diabetes, active tobacco use, obesity, and service location (inpatient and outpatient), to ensure comparison of similar surgical cases in a 1:1 ratio.

The primary outcome of interest was the average cost of CTR in the 90-day period related to the procedure. The cost was calculated as all costs, billed through CPT coding, incurred in the clinical work-up of the patient within 90 days before the operation, the procedure itself, and any postoperative costs such as therapy and clinic visits. These estimates typically do not include the facility fee, anesthesia charges, or other hospital-based costs. The secondary outcomes of interest were usage rates of EMG and nerve conduction studies (NCS) ordered in the 90 days prior to surgery, as well as the rate of therapy utilization within 90 days following surgery. In addition, complication rates, including postoperative infection and return to operating room, for infection were analyzed.

### Statistical analysis

Data were compiled and analyzed using PearlDiver software. Quantitative and qualitative evaluations of data were performed where appropriate. Demographics are provided as means and medians were appropriate. These costs are retrieved from Humana by looking at the reimbursement history recorded by Humana in PearlDiver. In addition, using the recorded date of the procedure as the setpoint, any other reimbursements made for a specific patient within +/− 90 days of the procedure can also be retrieved as a sum to create the total cost analysis. The average cost of clinical care was then compared using student *t* test; alpha was set at 0.05. Secondary outcomes were compared between orthopedic and plastic cohorts using chi-square analysis.

## Results

### Demographics

Over 2010–2022, a total of 1,236,256 patients treated with CTR were identified, with 497,671 treated by orthopedic surgeons, 76,130 treated by plastic surgeons, 59,240 treated by neurosurgeons, and 14,510 treated by general surgeons. After matching, each cohort totaled 13,107 patients. There were no significant differences in cohort characteristics based on matching factors, including age, sex, diabetes, obesity, tobacco usage, location of surgery, or Elixhauser Comorbidity Index (Table 1).

**Table 1:** Demographic of CTR Patients Separated by Provider Specialty

Characteristics	Orthopedics	Plastics	Neurosurgery	General Surgery	*P* Value
	N = 13107	N = 13107	N = 13107	N = 13107	
Women	8,464 (64.6%)	8,464 (64.6%)	8,464 (64.6%)	8,464 (64.6%)	
Diabetes	1,549 (11.8%)	1,549 (11.8%)	1,549 (11.8%)	1,549 (11.8%)	
Obesity	399 (3.0%)	399 (3.0%)	399 (3.0%)	399 (3.0%)	1
Tobacco use	636 (4.9%)	636 (4.9%)	636 (4.9%)	636 (4.9%)	
Inpatient	12 (0.1%)	14 (0.1%)	13 (0.1%)	11 (0.1%)	
Outpatient	13,042 (99.5%)	13,040 (99.5%)	13,078 (99.5%)	13,074 (99.5%)	
Other location	53 (0.4%)	53 (0.4%)	16 (0.4%)	22 (0.4%)	

### Comparison

There were statistically significant differences in the average total clinical cost. Plastic surgery had the highest mean clinical costs at $2,923, followed by neurosurgery at $2,923, orthopedics averaged $2,766, and general surgery averaged $2,607. Statistically, plastic surgery was equal to neurosurgery, which was both higher than orthopedic surgery, all three of which were greater than general surgery (*P* < .001). The distribution of open versus endoscopic CTR procedures varied by specialty, with open procedures comprising the majority of cases across all groups (plastics: 71%, orthopedics: 84%, general surgery: 79%, neurosurgery: 93%). Open CTR costs were significantly different among specialties (plastic = neurosurgery > orthopedics > general surgery; *P* < .001), whereas the mean endoscopic release costs did not differ significantly among any specialty (*P* > .05; Table 2).

**Table 2:** Mean Total Cost of CTR by Specialty

	Orthopedics	Plastics	Neurosurgery	General Surgery	*P* Value
Mean overall cost ± SD	$2,791 ± $3,895†	$2,934 ± $4,430‡	$2,988 ± $5,008‡	$2,613 ± $4,749∗	**<.001**
No. of open procedures	N = 10,953	N = 9,239	N = 12,076	N = 10,213	**-**
Mean open release cost ± SD	$2,768 ± $3,870†	$2,992 ± $4,759‡	$2,993 ± $5,004‡	$2,561 ± $4,743∗	**<.001**
No. of endoscopic procedures	N = 2,037	N = 3,738	N = 910	N = 2,734	**-**
Mean endoscopic release cost ± SD	$2,914 ± $4,024∗	$2,790 ± $3,482∗	$2,919 ± $5,051∗	$2,807 ± $4,765∗	**>.05**

Different superscript symbols indicate statistically significant difference between specialties as follows (*P* < .05).

Then, average cost of clinical care was compared using Student *t* test and alpha was set at 0.05. Bolded values represent statistical significance (*P* < .05).

∗ Significantly different from specialties marked with ^†^ and ^‡^.

† Significantly different from specialties marked with ∗ and ^‡^.

‡ Significantly different from specialties marked with ∗ and ^†^.

Usage rates of preoperative EMG and NCS utilization were highest by neurosurgeons at 28.5%, followed by plastics and orthopedics at 23.4% and 22.6%, respectively, with general surgeons ordering the fewest at 19.7% (*P* < .001 across specialties; Table 3). The rate of postoperative therapy ordered was the highest with plastic and orthopedic surgeons at 10.7% and 10.5%, respectively, followed by general surgery (7.9%), and neurosurgery (7.3%; *P* < .001 across specialties; Table 3).

**Table 3:** Rate of Perioperative Orders

Characteristic	Orthopedics	Plastics	Neurosurgery	General Surgery	*P* Value
	N = 13,107	N = 13,107	N = 13,107	N = 13,107	
Therapy	1,373 (10.5%)∗	1,401 (10.7%)∗	957 (7.3%)†	1,037 (7.9%)†	**< .001**
EMG and NCS	2,970 (22.6%)†	3,071 (23.4%)†	3,734 (28.5%)‡	2,581 (19.7%)∗	**< .001**

EMG, electromyography; NSC, nerve conduction studies.

Chi-squared analysis was used to analyze difference between rates.

Different superscript symbols and bolded values indicate statistically significant difference (*P* < .05):

∗ Significantly different from specialties marked with † and ‡.

† Significantly different from specialties marked with ∗ and ‡.

‡ Significantly different from specialties marked with ∗ and †.

Infection rates for neurosurgeons and general surgeons were statistically higher at 0.8% (n = 116) and 0.7% (n = 93), respectively, with orthopedic (n = 58) and plastic surgeons (n = 55) having a 0.5% infection (*P* = .002 across specialties; Table 4). There were minimal cases of return to operating room for infection for all specialties (<1%).

**Table 4:** Rate of Postsurgical Complications

Characteristic	Orthopedics	Plastics	Neurosurgery	General Surgery	*P* Value
	N = 13,107	N = 13,107	N = 13,107	N = 13,107	
Infection	68 (0.5%)∗	61 (0.5%)∗	109 (0.8%)†	94 (0.7%)†	.002
Return to OR for Infection	18 (0.1%)	<10 (-)	19 (0.1%)	<10 (-)	-

OR, operating room.

Chi-squared analysis was used to analyze difference between rates.

Different superscript symbols indicate statistically significant difference (*P* < .05).

∗ Significantly different from specialties marked with †.

† Significantly different from specialties marked with ∗.

## Discussion

Carpal tunnel syndrome is a common diagnosis seen by hand surgeons, which is frequently managed with surgical treatment by CTR. Overall, the specialty of hand surgery consists of surgeons from orthopedic, plastic, neurosurgery, and general surgery training backgrounds, with the vast majority of hand surgeons coming from either orthopedic or plastic surgery.^14^

Prior studies have analyzed the cost and rates of postoperative complications among these specialties. Baek et al^15^ found similar rates of postoperative complications for posterior lumbar fusions between orthopedic spine surgeons and neurosurgeons and higher costs for neurosurgeons. In the treatment of foot pathology, orthopedic surgeons performed significantly fewer procedures per episode and had significantly lower costs than podiatrists.^16^ Within the hand and wrist literature, previous work has shown differences in the rates of repair between orthopedic surgeons and plastic surgeons. Dasari et al^17^ found that orthopedic surgeons repaired carpal, radius, and ulna injuries on average 23.2 procedures per year compared with 2.6 procedures per year for plastic surgeons, which suggests that there may be volume or experience-based differences among specialties.

Unlike these studies, the current study demonstrates statistically significant, however, overall minimal and likely nonclinically significant differences among hand surgery providers in cost and utilization around CTR. This may be because of the longstanding integration of hand surgery fellowships, which commonly combine plastic, orthopedic, and general surgery, allowing for a sharing of techniques, skills, and knowledge among these specialties.^18^ Although there is significant overlap in specialties that perform hand procedures, there is currently a paucity of literature on the respective costs and postoperative complications. We sought to analyze CTR, one of the most frequent procedures seen by hand surgeons.^18^ Overall, the costs associated with treatment and rates of complications reported among the various subspecialty hand surgeons are the same clinically, despite the statistical significance noted among orthopedic, plastic, general, and neurosurgeons. We acknowledge that cost data are imperfect, and margins seen in this study may reflect billing differences, true cost differences, or implant/equipment discount differences. We aim to provide an objective numerical analysis, and readers may arrive at their own conclusions.

We found that, on average, plastic and neurosurgeons had the highest average total clinical costs related to treating CTS ($2,934 and $2,988, respectively), followed by orthopedics ($2,791), with general surgery having the lowest cost at $2,613 (*P* < .001; Tables 1 and 2).

However, it is difficult to determine the overall implications of this cost difference. Prior studies, such as Lowrance et al,^19^ found cost comparison differences of $255 and $296 between prostatectomy surgeries and concluded that there were no obvious cost implications. Similarly, we believe that although these results demonstrate a cost difference, its clinical significance is unclear. Future studies may be able to determine with more granularity the implications of these findings.

A major factor considered in our study was the location of service, inpatient versus outpatient, as studies have shown this to be a significant contributor to procedural costs.^13^ Our comparison cohorts had equal distributions of inpatient versus outpatient procedures, and as we expected, we found that CTRs are primarily outpatient procedures, with >99% of procedures being performed outpatient (Table 1).

In our study, the cost of care includes rates of associated services, including EMG/NCS and physical therapy. One explanation for the perceived differences in therapy prescriptions may be variations in practice patterns. Anecdotally, plastic surgeons and orthopedic surgeons are more likely to perform CTRs in practices with easy accessibility to hand therapy. We theorize that the small but appreciable differences in costs may reflect the slightly varying rates of preoperative and postoperative order utilization among specialties.

Additionally, we evaluated the influence of procedural technique (open versus endoscopic) on specialty-related cost differences. We found that the vast majority of procedures performed by all specialties were open releases, which drove the observed overall cost differences. Specifically, open release costs were significantly different among specialties (*P* < .001), mirroring overall cost differences, whereas endoscopic release costs were not statistically different across specialties (*P* > .05). This suggests that the observed variations in overall costs are predominantly influenced by differences in open CTR costs. The similar costs observed for endoscopic CTR across specialties reinforce that technique-specific factors may partially explain the subtle, yet significant, cost variations observed in our study. Future research could further explore the factors contributing to these specialty-specific differences, particularly within open procedures. (Table 2).

Although infection rates were overall quite low, and clinically speaking, similar, our results demonstrated that neurosurgeons and general surgeons had the highest rates of postoperative infection (0.8% and 0.7%) compared with orthopedic and plastic surgeons who had a postoperative infection rate of 0.5% (*P* < .001). The overall frequency of these events was extremely low, and the authors believe that the clinical differences are likely minimal. We also observed a low rate of patients returning to the operating room, with less than 1% of patients in each specialty having this complication. These events were rare, and small case counts prevented statistical comparison. These findings are consistent with existing literature reflecting low rates of postoperative infections reported by Harness et al,^20^ and Werner et al,^21^ at 1% and 0.32%, respectively. Overall, the key point here is that major complication rates, reflected as return to operating room, were uniformly low across specialties, whereas there were statistically significant differences in total complications across specialties. We speculate that the comparable outcomes seen in our study represent the common training fundamentals seen in combined hand fellowship programs. In a survey of hand fellowship directors, program directors strongly emphasized the treatment of wrist conditions as a core competency.^22^ In addition, hand fellows treat a similar number of nerve decompression cases during training.^23^

This study’s limitations mirror those of most database studies. With the data retrieved, we are fundamentally unable to link causation, but rather evaluate correlation, and are dependent on data quality. Database inputs are dependent on the accuracy of provider data input, which has a reported error rate of approximately 1.3%.^24^ In addition, because of the macroscopic nature of the database, our results do not account for other factors such as patient satisfaction, time to rehabilitation, or individual surgeon experience. In addition, making firm conclusions on the clinical implications of our cost findings is challenging. Future studies should explore factors such as cost granularity, rate of revision by specialty, surgeon experience, patient satisfaction, and more complex surgeries, such as flexor tendon repair, to further understand similarities and differences in the treatment of CTS. Finally, although there is controversy regarding technique, for example, open carpal tunnel technique versus endoscopic CTR, there is clinical value in understanding nationwide costs and trends in treatment of CTR and reflecting real-world variations in treatment approach.

In conclusion, the goal of this study was to compare the nationwide costs of CTRs performed by orthopedic, plastic, general, and neurosurgeons. We found that overall costs among specialists of hand surgery were not substantially different when evaluated for CTR. The statistically significantly greater cost per procedure performed by neurosurgeons and plastic surgeons is likely of minimal clinical significance and could be potentially explained by slightly differing rates of preoperative and postoperative order frequency. The main finding to be appreciated is that the four specialties analyzed perform CTRs with equal economic and clinical efficacy, reflecting common core fundamentals in treating wrist pathologies emphasized during training.

## Conflicts of Interest

Dr Strelzow is a consultant for Acumed, Stryker, and OrthoXel. The other authors have no relevant conflicts to disclose, and the authors declare that they have no known competing financial interests or personal relationships that could have appeared to influence the work reported in this paper.
